# MSDG: Multi-Scale Dynamic Graph Neural Network for Industrial Time Series Anomaly Detection

**DOI:** 10.3390/s24227218

**Published:** 2024-11-12

**Authors:** Zhilei Zhao, Zhao Xiao, Jie Tao

**Affiliations:** 1 School of Computer Science and Engineering, Hunan University of Science and Technology, Xiangtan 411201, China; 2 School of Mechanical Engineering, Hunan University of Science and Technology, Xiangtan 411201, China

**Keywords:** multi-scale sliding window mechanism, graph neural network, long short-term memory, multivariate sensor monitoring data, industrial equipment, spatial–temporal correlations, anomaly detection

## Abstract

A large number of sensors are typically installed in industrial plants to collect real-time operational data. These sensors monitor data with time series correlation and spatial correlation over time. In previous studies, GNN has built many successful models to deal with time series data, but most of these models have fixed perspectives and struggle to capture the dynamic correlations in time and space simultaneously. Therefore, this paper constructs a multi-scale dynamic graph neural network (MSDG) for anomaly detection in industrial sensor data. First, a multi-scale sliding window mechanism is proposed to input different scale sensor data into the corresponding network. Then, a dynamic graph neural network is constructed to capture the spatial–temporal dependencies of multivariate sensor data. Finally, the model comprehensively considers the extracted features for sequence reconstruction and utilizes the reconstruction errors for anomaly detection. Experiments have been conducted on three real public datasets, and the results show that the proposed method outperforms the mainstream methods.

## 1. Introduction

With the advancement of information technology, various sensors have been widely applied in status monitoring of industrial equipment, such as wind power equipment, computer server status monitoring, aerospace vehicles, machinery process control, etc. Sensors record the real-time operation of industrial equipment through diverse data, for instance, vibration, sound, temperature, and images, which together form multivariate sensor time series (MTS) [1]. In MTS, if there is a certain data segment deviating from the majority, it indicates that equipment may suffer damage or malfunction [2]. Once the staff fails to detect and deal with the anomalies in time, it is likely to cause equipment damage, machine shutdown, significant economic losses [3]. Therefore, establishing an efficient and stable model for anomaly detection in MTS is important, as it can ensure the safe and reliable operation of equipment and extend the equipment service life [4].

Current anomaly detection techniques fall into two main schools of thought: signal processing methods and machine learning-based methods [5]. Signal processing methods focus on analyzing the statistical properties of the data, often using techniques such as wavelet Gaussian, Hilbert transform and wavelet analysis to decompose the signal into its component frequencies. Mejia et al. [6] combined the wavelet multiresolution transform with robust prediction using a Gaussian process to process the data in the wavelet domain, using the transform function to capture geometric information and decompose it into simpler signals or subbands. Liu et al. [7] decomposed the shock signal by empirical modal decomposition and calculated the instantaneous frequency of the first eigenmode function, which characterizes the difference between the background noise and the shock signal. Karkhaneh et al. [8] used the wavelet transform to analyze non-stationary signals, then located abnormal states and highlighted abnormal curves in both time and frequency domains. These methods are particularly effective in identifying anomalies characterized by sudden changes in single-parameter variation. However, they have certain limitations, including a weak ability to handle nonlinear relationships and difficulty in detecting complex anomalous patterns [9]. On the other hand, machine learning-based methods use patterns learned from historical data to identify sequences that deviate from normal data. Commonly used techniques include clustering, autoencoders, and recurrent neural networks. Li et al. [10] used extended fuzzy clustering to reveal the structures present in the generated multivariate sub-sequences for anomaly detection. Yan et al. [11] proposed a hybrid convolutional autoencoder for machine tool anomaly detection under noise. Chander et al. [12] used a cascading recurrent neural network and a feature selection optimization algorithm for anomaly identification and classification. Chen et al. [13] used LSTM neurons and AE networks to construct a performance evaluation model for evaluating condition monitoring data from wind turbines. Kong et al. [14] constructed a generative adversarial network using bidirectional long short-term memory and an attention mechanism, which improves the accuracy of anomaly detection in industrial time series data. Longari et al. [15] developed an intrusion detection system based on an LSTM autoencoder that can detect anomalous activity in controller area networks. From these studies, it can be seen that machine learning methods can perform end-to-end anomaly detection on time series and learn complex structures and relationships from different types of MTS data [16]. Compared with traditional signal processing methods, machine learning methods are more suitable for mining multi-parameter relationships in MTS and can detect subtle anomalies in multidimensional temporal sequences.

However, industrial time series not only have temporal dependencies, but also spatial correlations between multivariate sensors. Graph Neural Network (GNN) is a machine learning model based on graph-structured data, which focuses on processing the complex relationship between nodes, edges and the whole structure, and can better extract the spatio-temporal correlation of MTS data. In GNN, multivariate sensor data are mapped to various nodes in the graphs, and the edges represent the relationships of nodes. GNN trains nodes and edges with historical data to capture correlations between variables in multidimensional data, which improved the accuracy and robustness of anomaly detection [17]. By modeling and learning potential correlations between sequences, GNNs demonstrate considerable potential for application in the MTS anomaly detection. Wang et al. [18] used GNN for decoupled representation learning to achieve anomaly detection in images. Song et al. [19] utilized two GNNs to extract spatial features and temporal correlations in MTS and identified performance anomalies affecting cloud environments. Han et al. [20] adopted GNN to extract graph-level features from cryptocurrency trading data and achieved anomaly detection of transaction time series.

The aforementioned methods primarily utilized GNN to represent complex relationships in multidimensional data, thereby improving the accuracy and robustness of anomaly detection. However, the correlations between various sensors will be changed over time. For example, wind turbine blades are driven by natural wind and then generate rotation. Sometimes, the data of the wind speed sensor are the same, but the data of the rotational speed sensor are variable, especially during the start-up and braking processes of wind turbines. Therefore, we will find that the correlation coefficient between the wind speed sensor and the rotational speed sensor will change at different times. Nevertheless, the existing research has generally adopted fixed-structure GNNs to directly extract the correlations between sensors, then obtained a fixed value correlation matrix, which struggles to capture the dynamic changes in the correlations between sensors. Secondly, the variation scale of time series also changes over time. For example, when the wind turbine runs smoothly, the variation scale in rotation speed is small, but during the braking processes, the variation scale in rotation speed is large. If the rotation speed changes are small, it is necessary to use a small-scale sliding window to extract features from the time sequence. When the speed changes greatly, it is necessary to use a large-scale sliding window to process the time sequence. Unfortunately, the existing GNNs often use the single-scale sliding window to extract features from time sequences. The sensory domains of GNNs are singular, so it is difficult to fully capture the correlation between sensors. For these reasons, this paper adopts the multi-scale sliding window mechanism to improve the GNN structure and combines LSTM to construct a multi-scale dynamic graph neural model (MSDG), so as to effectively extract the dynamic spatial–temporal correlations of MTS. The main contributions of this paper are as follows:

1. A multi-scale sliding window mechanism is introduced to segment MTS data at different temporal granularities, providing multiple perspectives for feature extraction.
2. A new module is proposed which adopts GNN and LSTM to construct a dynamic graph neural network and learn dynamic spatial–temporal correlations in MTS data.
3. The proposed method is evaluated on three publicly available datasets, and the effectiveness and contributions of each core component of the model are validated through ablation studies.

The rest of the paper is organized as follows. Section 2 introduces the concept of MTS anomalies and the current research status of anomaly detection algorithms based on GNN. Section 3 presents the MSDG model and indicates the functions of each component. Section 4 compares the method of this paper with existing methods on three publicly available datasets and analyzes the experimental results. Finally, Section 5 summarizes our work and points out future research directions.

## 2. Related Work

Graph Neural Network (GNN) represents the MTS data through graph structure. In GNN, nodes represent variables and edges represent the strength of association between nodes. GNN utilizes the information transfer mechanism and aggregation operation to allow nodes to exchange information with each other and update their states, capturing the interdependence between variables in multidimensional data, thus improving the accuracy and robustness of anomaly detection.

**Figure 1:**
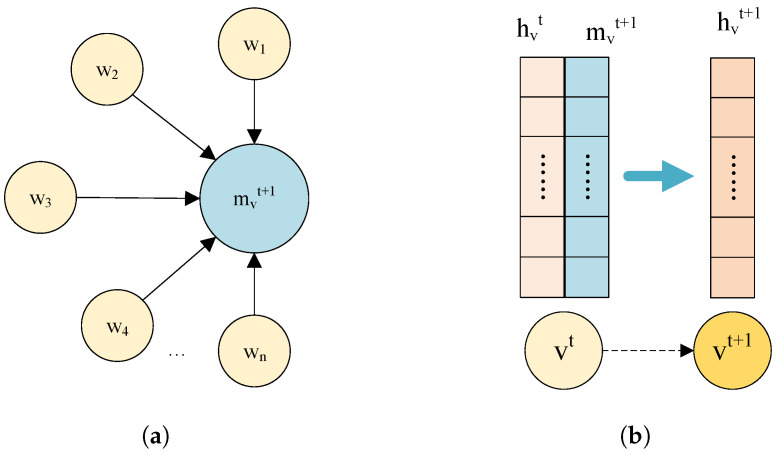
Fundamental structure diagram of GNN. (**a**) Aggregation; (**b**) Update.

GNN uses a message passing mechanism to aggregate information from neighboring nodes. Assuming that the graph G=(V,E) contains a set of vertices *V* and a set of edges *E*, where each vertex *v* has a feature vector $h_{v}$, the computational process of the message passing mechanism is shown in Figure 1a. For each node *v*, the message $m_{v}$ is first constructed based on its feature vector $h_{v}$ and the feature vector $h_{w}$ of the neighboring node *w*. The construction process can be carried out by a simple linear transformation or a more complex neural network.

(1) $$\begin{array}{c} m_{v}^{t+1}=\sum_{w\in N(v)}M_{t}\left(h_{v}^{t},h_{w}^{t},e_{vw}\right) \end{array}$$

where $m_{v}^{t+1}$ is the message received by node *v* at layer t+1, $M_{t}$ is the message function, $h_{v}^{t}$ denotes the node characteristics of node *v* in layer *t*, N(v) denotes the set of neighboring nodes of node *v*, $h_{w}$ denotes the node characteristics of neighboring node *w* of node *v* in layer *t*, and $e_{vw}$ denotes the characteristics of the edge from node *v* to node *w*.

Finally, the node features of the next layer of nodes $v^{t}$ are updated $h_{v}^{t+1}$, as shown in Figure 1b. This process can be realized by a nonlinear transformation. The formula is as follows:

(2) $$\begin{array}{c} h_{v}^{t+1}=U_{t}\left(h_{v}^{t},m_{v}^{t+1}\right) \end{array}$$

where $U_{t}$ is the node update function that takes as input the original node state $h_{v}^{t}$ and the message $m_{v}^{t+1}$ to obtain the new node state $h_{v}^{t+1}$.

To make better use of graph structure information, GNNs often include convolution, attention, or other components. These components enable GNN to efficiently process various types of graph data and show strong performance in anomaly detection. For instance, Chen et al. [21] computed the distance between all nodes and the global graph nodes, and filtered out the abnormal nodes by analyzing the offset of the distance of each node. Li et al. [22] used a graph neural network model by sparse adjacency matrix to reveal the interaction patterns in multiple time series channels, promote feature reconstruction between related channels, and improve the model’s anomaly detection performance. Furthermore, Huang et al. [23] adopted mutual information and graph embedding techniques to deeply learn and analyze complex data correlations in sensor networks, enabling them to accurately predict data changes at any point in time. Xu et al. [24] incorporated a random site masking strategy to systematically correct nodes or edges in the graph. This can improve the model’s ability to learn graph structure context and capture replacement data correlations. Zhang et al. [25] employed an autoencoder as the basic framework of GNN to construct a random relationship learning strategy, which comprehensively explored complex dependency relationships between sensors. Similarly, Guo et al. [26] proposed a sub-graph generation algorithm to construct a dependency graph with multiple sub-graphs’ corresponding centers, which explored the spatial correlation between the sensing data. Zheng et al. [27] used a graph neural network model based on a correlation-aware method to reveal the pairwise association properties among data. Chen et al. [28] decomposed key performance indicators into stable baseline trends and dynamic fluctuations. Then, they employed GNN to extract the intrinsic correlation patterns of MTS data in terms of attribute dimensions, entity correlations, and time series dynamics.

These studies showed that GNN has strong spatial modelling capabilities and can effectively capture the interdependencies between variables in MTS data. However, these methods fail to account for the limitations inherent in fixed graph structures and single-scale modeling, which hinders their ability to fully capture the dynamic characteristics within industrial time series. Particularly in practical engineering scenarios, where the time series data from equipment condition monitoring exhibit correlated variations in both time and space, there is a need for more advanced models to uncover the dynamic spatial–temporal features of MTS data. Therefore, this paper proposes the multi-scale dynamic graph neural model (MSDG) as a solution to the above problem. The model introduces a multi-scale sliding window mechanism to overcome the limitation of a single time scale. This mechanism is combined with a graph neural network to generate a corresponding graph structure for each time window, which dynamically evolves over time along with the correlations between sensor variables. The graph neural network is used to extract the correlation features for each time window separately, and then LSTM is used to model the dynamic evolution of the correlation features between time windows. Thus, the dynamic spatial–temporal correlation of the data is more comprehensively elucidated, and the ability of the model to capture and integrate information with different temporal resolutions is enhanced, which improves the performance of anomaly detection and generalization.

## 3. Methodology

**Figure 2:**
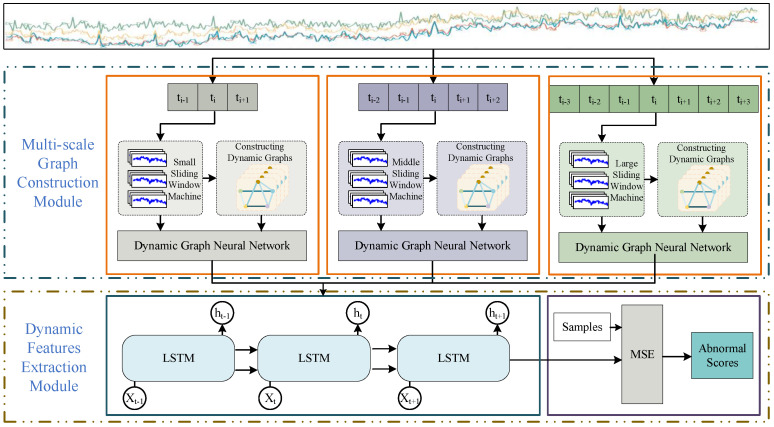
Structure of the MSDG model.

In order to fully reveal the dynamic spatial–temporal correlations embedded in MTS data, this paper proposes multi-scale dynamic graph neural network (MSDG) for industrial time series anomaly detection. As illustrated in Figure 2, the MSDG consists of two main components: a multi-scale graph construction module and dynamic feature extraction module.

Windows of different scales have receptive fields of various sizes [29]. In general, small windows are used to capture temporal transients, while large windows are used to extract stable features. In the multi-scale graph construction module, the MTS data are partitioned into time series segments with different window sizes. For example, the window size can be 3, 5, 7. With diverse window sizes, disparate sliding window mechanisms are formed to obtain multi-scale time segments. Then, multi-scale graph constructions were built with distinct temporal segments of raw MTS data. After that, the module represents the data segments as nodes in the graph, and adopts an attention mechanism to dynamically weigh the interactions between these nodes, thus constructing a series of dynamic graphs of node relationship evolution graph structure evolution. In the dynamic graph feature extraction module, we extract features from various time scales and use a concatenation strategy to fuse the dynamic graph features. Then, we utilize the powerful sequence modeling ability of LSTM to reconstruct the feature sequence data. Finally, the mean square error (MSE) between the original sequence and the reconstructed sequence is used to obtain the anomaly score of the data segment, which is used for anomaly detection of the time-series sequence.

### 3.1. Multi-Scale Graph Construction Module

This module mainly constructs multi-scale dynamic graphs through the multi-scale sliding window mechanism and the attention mechanism. The multiscale sliding window mechanism is designed to partition the data, creating distinct scales of data perspective. The attention mechanism learns the correlation between each variable in each window and transforms it into a node weight matrix.

**Figure 3:**
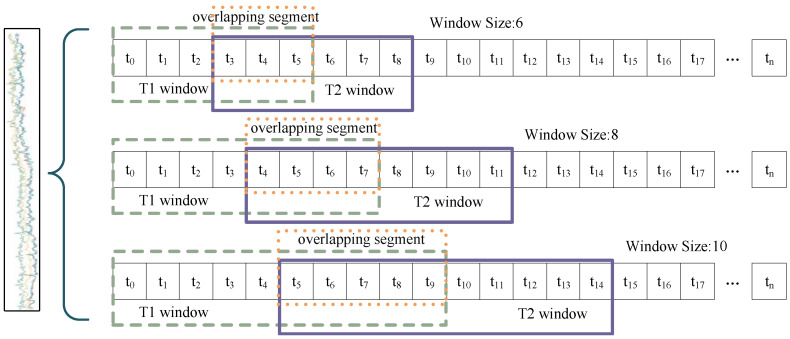
Schematic of multi-scale sliding window.

The multi-scale sliding window mechanism is shown in Figure 3; the left side of the curly brackets indicates a set of MTS data, and the right side indicates the partitioning of this set of data into three different windows. For an input data series $X=[x_{1},x_{2},\ldots ,x_{n}]$ with *n* time steps, which is partitioned using a sliding window, the data within the window can be represented as follows:

(3) $$\begin{array}{c} W_{i}=\left[x_{\left(i\cdot k\right)},x_{\left(i\cdot k+1\right)},\ldots ,x_{\left((i\cdot k)+w-1\right)}\right] \end{array}$$

where $W_{i}$ is the contained data sequence of the *i*th sliding window, *w* denotes the size of the sliding window, and *k* denotes the step size of the sliding window. The data after multi-scale sliding window segmentation can be expressed as follows:

(4) $$\begin{array}{cc} Z_{s_{j}} & =\langle W_{1},W_{2},\ldots ,W_{m}\rangle \end{array}$$

(5) $$\begin{array}{cc} Y_{s} & =\{Z_{s_{1}},Z_{s_{2}},\ldots ,Z_{C_{w,s}}\} \end{array}$$

where $Z_{s_{j}}$ represents the set of window sequences obtained following the segmentation of the data using the identical sliding window, and $C_{w,s}$ denotes the maximum number of instances in which a sliding window can be fully applied, given the window size *w* and the step size *s*. $Y_{s}$ represents the set of data obtained through the application of a multi-scale sliding window. The value of $s_{j}$ denotes the scale of the sliding window, while *m* represents the number of distinct sliding windows employed. For each window, the degree of association between variables is quantified by the attention mechanism. The attention mechanism is flexible enough to dynamically adjust the weight assignment based on the relative importance of the features at each position in the sequence [30].

Windows of multiple sizes allow for varying levels of temporal segmentation of the data [31]. The smaller windows provide more detailed temporal resolution. This makes them well suited for capturing short-term fluctuations and transient features. Such segmentation is more sensitive to changes in the data, and is particularly responsive to transient and rapidly changing features. Larger windows contain more localized information, thereby reducing the impact of noise and providing a more stable feature representation. The choice of window size depends on the time scale and granularity of the data being analyzed. Even after data segmentation, overlap between adjacent windows can preserve critical transition information or potential correlations. This effectively maintains data continuity and it is essential for capturing dynamic changes between data and identifying potential correlations. By integrating the dynamic spatial–temporal correlations in sliding windows of different scales, the model is able to synthesize the data features from a multi-scale perspective, which in turn improves the anomaly detection performance.

**Figure 4:**
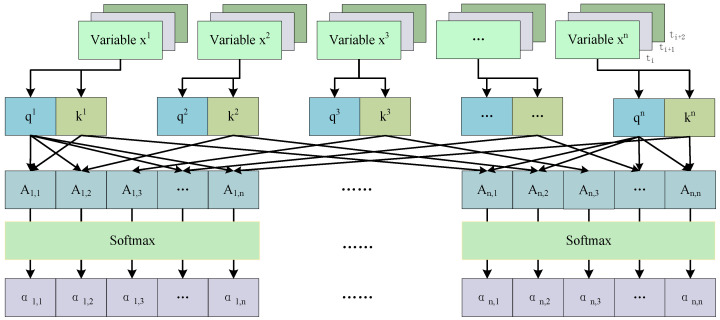
Attention mechanisms for constructing dynamic graph weight matrices.

The process of extracting the inter-node weight matrix by the attention mechanism is shown in Figure 4. The variable $x^{i}$ represents the temporal sequence segment of the ith variable within the specified window. For each time-series segment $X=\;<x^{1},x^{2},...,x^{n}>$ generated by a multi-scale sliding window, the following steps are undertaken. First, the query and key values of each variable are calculated; second, the attention score matrix is calculated; and finally, it is normalized by the softmax function to ensure that the resulting attention scores are transformed into weight distributions.

The data within the sliding window can be expressed as $x_{w}\in R^{T*N}$, where *T* denotes the number of time steps within the window and *N* denotes the number of variables. The mathematical expressions for query and key are as follows:

(6) $$\begin{array}{cc} Q & =W^{Q}x_{W}+b^{Q} \end{array}$$

(7) $$\begin{array}{cc} K & =W^{K}x_{W}+b^{K} \end{array}$$

where $W^{Q}$ represents the weight matrix utilized to compute *Q*, $W^{K}$ denotes the weight matrix employed to compute the key *K*, and $b^{Q}$ and $b^{K}$ are the bias terms associated with both, respectively. The attention score can be obtained through a dot-product operation, and its mathematical expression is as follows:

(8) $$\begin{array}{c} A=\frac{QK^{T}}{\sqrt{d_{k}}} \end{array}$$

where *A* represents the matrix of attention scores among variables, while $\sqrt{d_{k}}$ denotes the dimension of the key vector. This vector is employed as a scaling factor to effectively inhibit the numerical inflation that arises from the inner product of high-dimensional vectors. Furthermore, this approach facilitates the trainability of the gradient of the softmax function, thereby enhancing the overall performance of the model. Subsequently, softmax operations are conducted on all variables to obtain the normalized attention weights. The formula is as follows:

(9) $$\begin{array}{c} \alpha =softmax(A) \end{array}$$

where the symbol α denotes the normalized attention score. The value of α is then used as the weight matrix of the local graph in the subsequent modeling process. This essentially reflects the relative importance of different elements or the degree of attention allocation. In this manner, each node is assigned a weight based on the normalized attention score α, which accurately reflects its contribution to the overall information interaction. In summary, the normalized attention score α can represent the spatial dependence of MTS data variables, and the continuous score constructed in a continuous window can represent the dynamic spatial–temporal dependence of MTS data variables.

**Figure 5:**
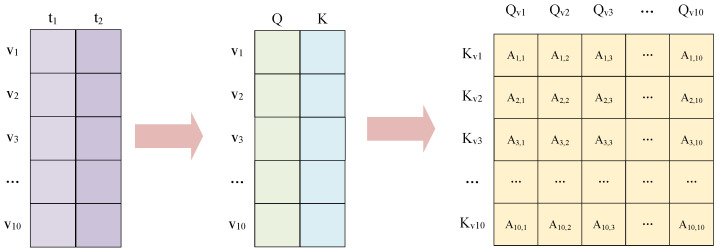
The process of generating weight matrices for the graphical structure of wind turbine datasets.

Shown in Figure 5 is the process of generating the procession of a graph structure weight matrix for a wind turbine dataset, which contained 10 variables. The sliding window size is 2. The purple matrix in the figure is a 10 × 2 matrix that stores ten variable data points at time $t_{1}$ and ten variable data points at time $t_{2}$. Secondly, the query and key matrices are computed based on Equations (6) and (7). And the results of the calculations are stored in column *Q* and column *K* of the intermediate matrix in the figure. Then, the weights between the variables are calculated using Equation (8) to form a 10 × 10 matrix. Finally, these weights are normalized by the softmax function to obtain a 10 × 10 matrix of weights.

### 3.2. Dynamic Graph Feature Extraction Module

In the dynamic graph feature extraction module, we use GNN and LSTM to extract dynamic features, which consists of three phases: local feature extraction, spatial correlation feature extraction and temporal correlation feature extraction. The objective of the local feature extraction phase is to identify the node features that constitute the fundamental units of the graph, focusing on the direct neighborhood of each node. The spatial correlation feature extraction stage involves the iterative propagation and updating of node features. This process considers not only the node’s intrinsic attributes but also integrates the transformed information propagated from neighboring nodes. This approach facilitates the diffusion of node representations and information fusion within the graph [29]. The temporal correlation feature extraction component is concerned with the identification of dynamic patterns in graph structure and node features that evolve over time in a series of data points. The combination of these three components enables the dynamic graph neural network to effectively learn the spatial–temporal correlations of graph data. It is able to not only capture transient graph structural features but also to model dynamic spatial–temporal correlations.

**Figure 6:**
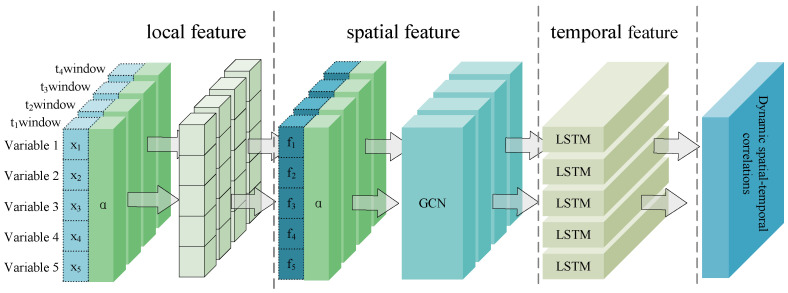
Dynamic graph neural network for dynamic spatial–temporal correlation feature extraction.

As illustrated in Figure 6, the graph node represented by *x* corresponds to the MTS variable, while the graph structure weight matrix represented by α corresponds to the window data at each moment. First, this paper capture the local features of the short-term data within each window. Subsequently, the refined local features are combined with their corresponding graph weight matrices and fed into a dynamic graph neural network model, thereby uncovering dynamic spatial–temporal correlations that are hidden in the time series. The GNN is responsible for learning the correlation between sensor variables in each time window independently. The LSTM is responsible for integrating the correlations in each time window and modeling the process of correlation change in successive time windows. These two components work in conjunction to achieve the effective fusion of spatial–temporal features. Dynamic Graph Feature Extraction can be expressed by the following equation:

(10) $$\begin{array}{cc} L^{t} & =f(x^{t}) \end{array}$$

(11) $$\begin{array}{cc} Z^{t} & =GNN(G^{t},L^{t}) \end{array}$$

(12) $$\begin{array}{cc} H^{t} & =LSTM(H^{t-1},Z^{t}) \end{array}$$

where $L^{t}$ denotes the local features of the data at time *t*, *f* denotes the local feature extraction function, $x^{t}$ denotes the function at time t, $G^{t}$ denotes the graph structure at time t, $Z^{t}$ denotes the spatial features extracted by GNN, and $H^{t}$ denotes the dynamic spatial–temporal features integrated by LSTM at time t. $H^{t-1}$ denotes the dynamic spatial–temporal features at the previous time.

Through sliding windows of three different sizes, we obtained three matrices: $H_{1}$, $H_{2}$ and $H_{3}$. Then, we used cascade operations to transform the three matrix values into a feature matrix, and the feature matrix is fed into the LSTM, which outputs the reconstructed signal. For the original sample *x* and the reconstructed sample $\hat{x}$, we calculate its mean square error (MSE) as the anomaly score for that sample, where $L_{Score}$ is the anomaly score.

(13) $$\begin{array}{c} L_{Score}=MSE\left(x,\hat{x}\right)=\frac{1}{mn}\sum_{i=1}^{m}\sum_{j=1}^{n}{\left(x-\hat{x}\right)}^{2} \end{array}$$

The dynamic graph feature extraction module captures the dynamic patterns that evolve over time in graph data by combining the extraction process of local features, spatial correlation and temporal correlation. The dynamic graph neural network constructed by using GNN and LSTM can not only extract the instantaneous features of the graph structure, but also model the complex spatial–temporal correlations. The module achieves a comprehensive characterization of node and structure changes in dynamic graphs, thus improving the detection of temporal anomalies.

## 4. Experimental Studies

In this section, we present the experimental dataset and performance metrics used in our studies. Then, our method is compared with four mainstream methods to demonstrate the effectiveness of MSDG. In addition, we show ROC curves for each model to visually compare their performance. Finally, we also perform an ablation study to validate the necessity of each component in the MSDG model, and further demonstrate its robustness and reliability.

### 4.1. Datasets

In this paper, we utilize three publicly accessible datasets, including WT23 [32], SMD [33], and PSM [34], and conduct pertinent experiments on the aforementioned datasets. WT23 is a publicly available wind turbine dataset. This dataset is derived from real-time data recorded by sensors in actual operating wind turbines. The monitoring data of wind turbine status is very large, and we have selected seven sensor data points that are most relevant to equipment anomalies for experimentation. The SMD dataset comes from the equipment information of a large internet enterprise, including the running status records of 28 machines, which are divided into three various entity groups. Due to the differences in the operating environment of each machine, these 28 subsets should be trained and tested separately. For each subset, the data are divided into two equal-length parts, which are used for training and testing, respectively. In this paper, one of the subsets is selected for training and testing. PSM is a dataset comprising pooled server metrics, collated from a multitude of application server nodes, and encompassing 26 distinct feature variables. These features describe metrics related to the operation of the server machine, including CPU utilization and memory usage. PSM is a dataset for monitoring the operational status of computer servers, containing 26 different feature variables. These variables describe indicators of the operating status of server devices, such as CPU utilization and memory usage. The abnormal situations in the PSM test set were manually marked by engineers and application experts. We selected 13 weeks of data to train the model and used an 8-week dataset as a test set to evaluate the accuracy and generalizability of the model’s predictions. Table 1 provides a comprehensive account of the dataset, offering detailed information on each dataset. Among them, WT23, SMD, and PSM had anomaly rates of 29.80%, 9.46%, and 27.76%.

The WT23, SMD, and PSM datasets are manually divided into training sets and test sets. The training sets are assumed to represent normal data. The test sets comprised the data in system abnormal conditions. The data in each dimension of the dataset are subjected to min-max normalization, whereby the values of the data are mapped to the interval [0,1].

### 4.2. Evaluation Metrics and Comparison Methods

This paper employs three commonly utilized evaluation metrics to assess the efficacy of time series anomaly detection, namely precision, recall, and F1-score. Precision refers to the proportion of actual positive classes among the samples predicted to be positive, and thus measures the accuracy of the model in predicting positive classes. Recall is the proportion of correctly predicted positive classes among all the samples with positive classes, and thus measures the model’s detection rate of positive classes. F1-Score is the harmonic mean of precision and recall, and thus is able to consider the model’s accuracy and detection rate together. They are calculated as follows:

(14) $$\begin{array}{cc} Precision & =\frac{TP}{TP+FP} \end{array}$$

(15) $$\begin{array}{cc} Recall & =\frac{TP}{TP+EN} \end{array}$$

(16) $$\begin{array}{cc} F1-score & =\frac{2\times Precision\times Recall}{Precision+Recal} \end{array}$$

where TP represents a true positive, FP denotes a false positive, and FN signifies a false negative. The values of precision, recall, and F1-score range from 0 to 1, with a value of 1 indicating optimal performance. In this paper, all methods use the optimal threshold search strategy to obtain the anomaly threshold.

In this study, we evaluate the efficacy of our proposed method for anomaly detection in graphs by comparing its performance with four popular graph neural network-based methods.

MTAD-GAT [35] is a graph attention network-based method. It treats each univariate time series as a separate feature, extracts spatial and temporal features through two graph attention layers in parallel, and reconstructs and predicts the time series after fusing the temporal and spatial features.

STGAT [32] coherently captures features and temporal correlations between MTS data through a stackable spatial–temporal graphical attention network. Temporal correlations at different time scales are captured by a multi-scale input network. The extracted features are employed for the reconstruction of the time series, which are then classified based on the reconstruction effect.

ATCN [36] uses dynamic graph attention to model the complex correlation between variables and time series. Then, a temporal convolutional network with parallel processing capability is utilized to extract multidimensional features for downstream prediction tasks. Finally, the prediction error is used to detect anomalies.

GDN [37] integrates structure learning and attention mechanisms to provide interpretability for anomaly detection in MTS data. This work proposes a graph deviation network approach, which attempts to capture the distinctive features of each variable and construct a graph of relationships between variables using embedding vectors. Ultimately, the GDN detects anomalies through the prediction bias of the graph attention function.

### 4.3. Result and Analysis

This paper conducts comparative experiments between the MSDG model and the graph neural network-based models, such as MTAD-GAT, STGAT, ATCN, and GDN methods. The parameter set times of MSDG are mainly determined by the number of sensors in the dataset. The WT32 dataset contains monitoring data from ten sensors, so the model has 10 nodes and 90 edges. The SMD dataset contains 38 types of sensor monitoring data, so the model has 38 nodes and 1406 edges. The PSM dataset contains 25 types of sensor data, so the model has 25 nodes and 600 edges. Based on experience, in the WT32 and SMD experiments, the sizes of the multi-scale sliding windows are 1, 2 and 3. In the PSM dataset, the changes in the sensor data are relatively small, so multi-scale time windows of 2, 4 and 6 are used. The other four model parameters are consistent with their descriptions in the literature. The results of these experiments are presented in Table 2.

On the WT23 dataset, the precision of the MSDG is 85.77%, the recall rate is 93.49%, and the F1-score is 0.89. All three results are superior to the other four models. On the SMD dataset, MSDG has the highest precision, reaching 65.35%, and the F1-score also reaches 0.71. The recall rate of GDN is the best at 75.98%, but only 0.1% higher than MSDG. Due to the complexity of PSM data, the precision of all five models is not high. The precision of MSDG is 55.82%, while the accuracy of the other four models is only about 45%. The SMD dataset is more imbalanced than the WT23 and PSM datasets, and MSDG has a higher dimensionality than the WT23 and PSM datasets. Consequently, our method demonstrates effectiveness even in unbalanced and high-dimensional attack scenarios, which are of significant importance in real-world applications.

MTAD-GAT employs GNN to elucidate the spatial correlation among MTS data and integrates time series attributes to achieve effective modeling of MTS data. However, this approach cannot adequately account for the dynamic spatial–temporal correlations inherent in MTS data, which affects the model’s ability to identify anomalous events.

In contrast, STGAT employs a multi-scale input strategy that captures the temporal correlation of MTS at various time scales. Concurrently, this approach integrates temporal and spatial correlations through the architectural configuration of a stacked graph attention network. Although the STGAT method does not explicitly model the dynamic spatial–temporal correlations inherent in MTS data, its ability to capture these correlations across different time scales helps to partially mitigate this disadvantage. Compared to the MTAD-GAT, the STGAT model exhibited superior performance on three independent datasets.

The ATCN uses a dynamic graph attention mechanism that captures complex correlations between variables and time steps, while its weighted embedding operation enhances the design of its dynamic graph architecture. This strategy enables ATCN to achieve more outstanding performance on the WT23 and SMD datasets. However, the model is still limited by a single time scale, with an accuracy of only 41.28% and a recall of 0.52 on the PSM dataset. The performance of this model on other datasets is also unsatisfactory.

GDN proposes a graph deviation network architecture aimed at extracting unique representations of each variable. It constructs a graphical relational model that reflects the interactions between the variables using embedding vector techniques. The method predicts future state changes for each sensor by applying an attention mechanism to neighboring sensors within the graph. Additionally, it identifies potential biases in the structure of relationships between sensors, which are learned based on the graph bias score. Similarly, the GDN method is not designed to adequately account for the dynamic spatial–temporal correlations of the variables in the MTS data as they evolve over time. This limitation constrains the model’s capacity to capture the complex dynamic correlations within MTS data during an anomaly detection task.

MSDG initially employs the sliding window technique to segment the MTS data, thereby capturing local features across various time scales. Subsequently, it constructs dynamic graphs that reflect the intrinsic spatial–temporal correlations of the data. This approach allows a multifaceted perspective to be taken with regard to the extraction of features. Secondly, MSDG employs dynamic graph neural networks to analyze the characteristics of the spatial–temporal correlations implicit in MTS data within these dynamic graphs. This process can be described as the modeling of dynamic spatial–temporal correlations. Ultimately, to integrate long-term temporal correlation data, the method incorporates an LSTM, which effectively captures and models long-term temporal series dependencies in MTS data, thereby reconstructing the original MTS data on this basis. The MSDG is capable of modeling the intricate spatial–temporal structure and dynamic evolution of MTS data, thus facilitating enhanced anomaly detection.

**Figure 7:**
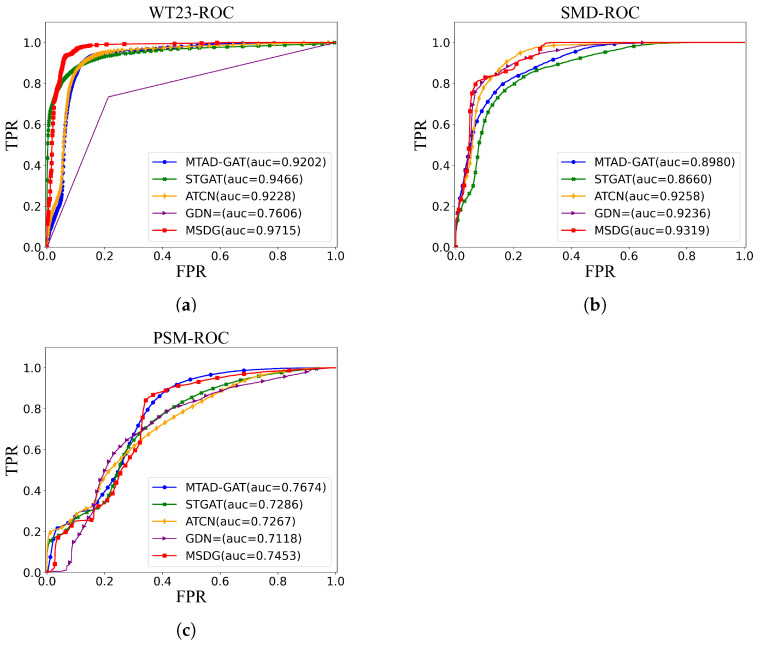
ROC curves for model performance on three datasets. (**a**) WT23-ROC; (**b**) SMD-ROC; (**c**) PSM-ROC.

To further validate the model performance, this paper presents the ROC-AUC plots of the four methods in Figure 7. The vertical axis represents the true positive rate, and the horizontal axis represents the false positive rate. The ROC curve illustrates the correspondence between the true positive rate and the false positive rate of each method in the dataset, and the area under the ROC curve is the AUC. A higher value of AUC indicates a superior model performance. Figure 7a shows the WT23-ROC curves, and it can be seen that the GDN model has the worst performance, with an AUC value of 0.7606. Conversely, the MSDG model has the best performance, with am AUC of 0.9715, and the other models are positioned between these two extremes. Figure 7b shows the SMD-ROC curve, and it is observed that the STGAT model has the worst performance, characterized by an AUC value of 0.8660. The MSDG model stands out with the best performance, achieving an AUC of 0.9322. Figure 7c shows the PSM-ROC curve, and the MTAD-GAT model has improved its performance, achieving an AUC value of 0.7674. The MTAD-GAT model ranks second after the MSDG model, which has an AUC value of 0.7453. Overall, the performance of the MTAD-GAT model varies widely across datasets and is generally poor. The STGAT model demonstrates greater consistency, although it does not excel in all three datasets. The GDN model performs well on the SMD dataset, but performs poorly on the WT23 and PSM datasets. MDGN shows better performance in all three tasks. This implies that the MSDG model performs well in terms of false positive and true positive rates at various preselected thresholds, which is important for practical applications.

### 4.4. Ablation Study

**Figure 8:**
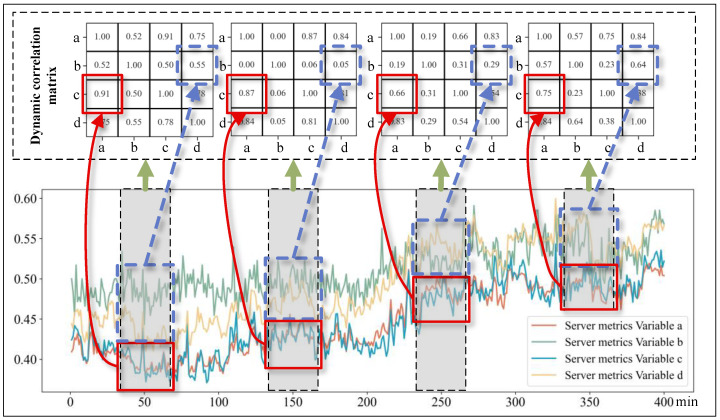
Example of dynamic spatial–temporal correlations of four variables in the computer server indicator dataset.

In order to verify the validity and contribution of the components within the MSDG model, a series of ablation experiments are designed and implemented. As shown in Figure 8, the graph shows 400 min of anonymous metric data from a computer server.

Although the exact meta-attribute details of the variables are inaccessible, we can still plot line graphs for several time-series variables to examine their interrelated patterns of change. In the figure, the x-axis represents time, while the y-axis represents the measurement ranges of each variable. Green arrows indicate the association of the correlation matrices with these gray periods. The two blue squares connected by a blue arrow represent the correlation between variables a and c along with their respective fluctuations, while the two red squares connected by a red arrow represent the correlation between variables b and d along with their respective fluctuations. Specifically, the correlation coefficient between variables a and c is 0.91 within the initial observation window (35–65 min), decreases to 0.87, 0.66, and then increases again to 0.75 in subsequent comparable intervals (135–165, 235–265, and 335–365 min). This pattern vividly illustrates the fluctuating nature of inter-variable correlations over time. Similarly, the correlation coefficients of variables b and d are 0.55, 0.05, 0.29, and 0.64 in that order, exhibiting dynamic changes over time. Therefore, Figure 8 showcases the dynamic spatial–temporal correlations among variables in MTS data over time.

To further understand the contribution of each component within the MSDG model, two variant models of the MSDG are tested. In the first variant, the model is named MSDG-SingleScale, which removes the multi-scale sliding window structure and uses a single time scale as the basis for dynamic graph construction. In the second variant, the dynamic graph generation module is removed and replaced with a fixed static graph structure, which is input to GNN for processing. This version is labeled MSDG-SingleGraph. The optimal F1-scores achieved by these three configurations of the model are shown in Table 3.

As shown in Table 3, on the WT23 dataset, the F1-score of MSDG is 0.8886, while MSDG-SingleScale achieves only 0.8377, and the other variant, MSDG-StaticGraph, achieves only 0.8201. On the SMD dataset, the F1-score of MSDG is 0.7125, while the F1-scores of the two variants are only 0.5044 and 0.5578. On the PSM dataset, the F1-score of MSDG is 0.6733, while the two variants achieve only 0.5298 and 0.5825. The MSDG model achieves the optimal F1-scores on these three different datasets, mainly due to its effective modeling of multi-scale local features and dynamic spatial–temporal correlations. In contrast, the MSDG-SingleScale model may not effectively extract multi-scale local features. It focuses solely on a single time step when constructing the dynamic graphs and ignores the spatial correlation information among multiple time steps. Consequently, it cannot accurately construct the dynamic graphs that reflect the intrinsic dynamic relationship of the MTS data. On the other hand, although the MSDG SingleGraph model captures the spatial–temporal correlations of MTS data through a fixed graph structure, it ignores the spatial–temporal correlation characteristics over time, resulting in poor performance in capturing dynamic spatial–temporal correlations. Therefore, the MSDG model achieves the best performance in evaluation metrics by integrating and optimizing the modeling capabilities of multi-scale local features and dynamic spatial–temporal correlations.

### 4.5. Analysis of Window Size Parameters

To investigate the effect of sliding windows of different scales on the performance of MSDG models, we conducted experiments on three MTS datasets: WT23, SMD and PSM. First, model performance was evaluated under a single-scale sliding window (the sizes ranging from 1 to 9). Meanwhile, we have designed nine multi-scale sliding windows for anomaly detection experiments.

As shown in Table 4, we conducted nine experiments on three datasets, all the experiments in the single-scale sliding window mechanism. The window sizes are introduced in the first column. The average F1 scores of the three datasets were 0.8454, 0.5041, and 0.5591, respectively. Among them, the highest value of the WT32 experiment appears in window 7. The highest value of the SMD experiment appears in window 2. The highest value of the PSM experiment appears in window 8. This indicates that the different datasets are suitable for different sliding window sizes. If the model adopts a single sliding window mechanism, it will limit the generalization ability of the method.

Since the MSDG model relies on a sliding window to generate a dynamic graph structure, which in turn captures the dynamic evolution of the correlations between the variables in the MTS data, the size of the sliding window should be set to a smaller value to capture the dynamic correlations in a more detailed way. Also, ideally, the window size should be small enough to capture transient features and large enough to generate smooth joint features. Therefore, we empirically selected nine different multi-scale sliding window configurations to discuss the effect of multi-scale sliding window size on the anomaly detection performance of the MSDG model.

Table 5 shows the results of the multi-scale sliding window experiments. We designed nine sets of multi-scale sliding window experiments, with specific scale settings as shown in the first column of Table 5. We conducted experiments on three datasets and the average F1 scores were 0.8501, 0.6766 and 0.6497, respectively. The results were superior to the single-window mechanism in Table 4, indicating that multi-scale sliding window mechanisms can effectively improve the feature extraction ability of the model and more comprehensively capture abnormal states in MTS. For example, for the WT23 dataset, the model achieves the best performance (F1 score = 0.8886) when using the ‘1, 2, 3’ multi-scale configuration. This suggests that by combining information from different time scales, the model is able to understand the temporal patterns more comprehensively, thus improving the accuracy of anomaly detection. The SMD and PSM datasets show similar trends, with the SMD showing the most significant performance improvement in the ‘1, 4, 7’ configuration, while the PSM performs better in a variety of multi-scale configurations, particularly in the ‘2, 4, 6’ window scale configurations. This suggests that the window configurations are not the same for different datasets to achieve the best performance, but individual datasets perform well when the window scales are small, so smaller combinations of window scales, e.g., ‘1, 2, 3’, can be considered for practical applications. This is because the MSDG model requires the use of sliding windows to simulate the dynamic evolution of correlations between variables, and larger windows tend to obscure the details of the evolution of these correlations.

## 5. Conclusions

This article adopts the multi-scale sliding window mechanism and LSTM to improve the GNN, and it constructs a new multi-scale dynamic graph neural network (MSDG) for industrial equipment anomaly detection. And the MSDG can capture the dynamic spatial–temporal correlations among high-dimensional sensor data. To verify the performance of MSDG, three publicly available device status monitoring datasets were selected for anomaly detection experiments. Compared with traditional graph neural networks, the results of the experiments showed that MSDG achieved the highest F1-score, and the accuracy increased by 0.45%, 11.47% and 7.67%, respectively, and our recall rates reached 93.49%, 75.85% and 84.05%, respectively. These experimental results show that the MSDG algorithm can achieve better anomaly detection and can be applied to anomaly detection in multidimensional time series.

## Figures and Tables

**Table 1:** Statistics of the three datasets used in experiments (units: number).

Dataset	Features	Train	Test Normal	Test Anomaly
WT23	10	67,090	16,491	7003
SMD	38	28,479	25,785	2694
PSM	25	132,481	63,460	24,381

**Table 2:** Performance comparison of different methods and datasets (P: Precision, R: Recall, F1: F1-Score).

	WT23			SMD			PSM		
Method	P(%)	R(%)	F1	P(%)	R(%)	F1	P(%)	R(%)	F1
MTAD-GAT [35]	75.73	89.77	0.82	47.48	53.56	0.50	45.75	87.37	0.60
STGAT [32]	85.32	81.96	0.84	45.30	66.35	0.54	48.15	81.21	0.60
ATCN [36]	77.78	86.93	0.82	48.12	74.61	0.59	41.28	71.75	0.52
GDN [37]	58.13	73.41	0.65	53.88	75.98	0.63	42.31	7813	0.55
MSDG (our method)	85.77	93.49	0.89	65.35	75.85	0.71	55.82	84.05	0.67

**Table 3:** Optimal F1-Score for MSDG model ablation experiments.

Method	WT23	SMD	PSM
MSDG-SingleScale	0. 8377	0. 5044	0. 5298
MSDG-StaticGraph	0. 8201	0. 5578	0. 5828
MSDG (our method)	0. 8886	0. 7125	0. 6733

**Table 4:** The F1 score of MSDG model performance in single window.

Single Window Scale	WT23	SMD	PSM
1	0.8654	0.5043	0.5303
2	0.8224	0.5102	0.5298
3	0.8201	0.4993	0.5291
4	0.8382	0.5033	0.5294
5	0.8330	0.5097	0.5297
6	0.8526	0.5080	0.5284
7	0. 8940	0.5042	0.5298
8	0.8304	0.4981	0.5804
9	0.8439	0.5001	0.5721
Avg.	0.8454	0.5041	0.5591

**Table 5:** The F1 score of MSDG model performance in multiple windows.

Multi-Window Scale	WT23	SMD	PSM
1, 2, 3	0.8886	0.7125	0.6733
1, 3, 5	0.8259	0.7062	0.6510
1, 4, 7	0.8758	0.7144	0.5660
2, 3, 4	0.8445	0.6965	0.6632
2, 4, 6	0.8491	0.6364	0.6744
2, 5, 8	0.8485	0.6071	0.6308
3, 4, 5	0.8514	0.6722	0.6700
3, 5, 7	0.8401	0.6553	0.6548
3, 6, 9	0.8268	0.6887	0.6634
Avg.	0.8501	0.6766	0.6497

## Data Availability

The WT23 dataset is made available to the public by its authors and can be accessed at this URL: https://github.com/zhanjun717/STGAT (accessed on 12 September 2024). The SMD dataset is made available to the public by its authors and can be accessed at this URL: https://github.com/NetManAIOps/OmniAnomaly (accessed on 12 September 2024). The PSM dataset is made available to the public by its authors and can be accessed at this URL: https://github.com/eBay/RANSynCoders (accessed on 12 September 2024).
